# Developing type 1 diabetes resources: a qualitative study to identify resources needed to upskill and support community sport coaches

**DOI:** 10.3389/fcdhc.2023.1284783

**Published:** 2023-11-01

**Authors:** Rachel J. Lim, Alison G. Roberts, Joanne M. O’Dea, Vinutha B. Shetty, Heather C. Roby, Elizabeth A. Davis, Shaun Y. M. Teo

**Affiliations:** ^1^ Rio Tinto Children’s Diabetes Centre, Telethon Kids Institute, The University of Western Australia, Perth, WA, Australia; ^2^ Department of Endocrinology and Diabetes, Perth Children’s Hospital, Perth, WA, Australia; ^3^ Division of Paediatrics within the Medical School, The University of Western Australia, Perth, WA, Australia; ^4^ Centre for Child Health Research, The University of Western Australia, Perth, WA, Australia

**Keywords:** type 1 diabetes, community sport, resources, knowledge, confidence, education

## Abstract

**Introduction:**

Community sport coaches in Western Australia lack an understanding, the confidence, and knowledge in supporting young people with Type 1 diabetes (T1D). This study aims to identify what T1D educational resources are required to upskill coaches in Western Australia.

**Methods:**

Semi-structured online interviews were conducted with i) young people living with T1D, ii) parents of young people living with T1D and iii) community sport coaches. The questions explored i) past experiences of T1D management in community sport ii) the T1D information coaches should be expected to know about and iii) the format of resources to be developed. Thematic analysis of interview transcripts was performed, and the themes identified were used to guide resource development.

**Results:**

Thirty-two participants (16 young people living with T1D, 8 parents, 8 coaches) were interviewed. From the interviews, young people wanted coaches to have a better understanding of what T1D is and the effect it has on their sporting performance, parents wanted a resource that explains T1D to coaches, and sports coaches wanted to know the actions to best support a player living with T1D. All groups identified that signs and symptoms of hypoglycaemia and hyperglycaemia needed to be a key component of the resource. Sports coaches wanted a resource that is simple, quick to read and available in a variety of different formats.

**Conclusion:**

The interviews resulted in valuable information gained from all groups and have reinforced the need for the development of specific resources to increase community knowledge and provide support for players with T1D, parents and sport coaches.

## Introduction

The majority of physical activity (PA) that Australian children engage in is through organised sport outside of school hours (1). In Australia, community sport is one of the most common settings in which children and young people exercise, with approximately 71% of children between the ages of 5-14 years participating in some form of organised sport (2). There are numerous known health benefits of PA, however, additional health benefits of PA exist for people living with type 1 diabetes (T1D). These include improvements in glycaemic control (3), blood lipid profiles, cardiovascular function (4, 5), and enhanced psychological wellbeing (6). Despite these associated PA benefits being widely recognised, levels of PA remain low, with only 23% of Australian children aged 5-14 years meeting the total recommended levels of 60 minutes of physical activity per day (1). More importantly, the percentage is much lower in young people living with T1D (7). These low PA levels observed in children living with T1D can lead to a potential lost opportunity to minimise cardiovascular disease risk and burden of disease that is associated with T1D.

A plausible reason for the lower rate of PA in young people with T1D is that exercise management for T1D is complex. This complexity is reflected through the numerous factors that influence an individual’s glycemic responses to exercise, including exercise intensity, duration, and timing of exercise in relation to insulin administration (8). Consequently, the key to achieving a safe and effective exercise management plan is providing exercise recommendations (8, 9). Previous research by our team has identified a key challenge faced by youth living with T1D is a lack of awareness, understanding, and support surrounding diabetes management recommendations around exercise (10) and is one of the many barriers to participation in PA (11), particularly in community sport. In addition, focus groups with players living with T1D revealed that coaches had poor knowledge, awareness and understanding surrounding T1D and this was a barrier to player’s participation and performance in community sport (10). Similarly, previous studies have reported the difficulties young people experience with their physical education teachers who often held them back from participating in certain sporting activities due to i) fear of what might happen, ii) being overly cautious or iii) misunderstanding the limitations of diabetes and the effect it has on their ability to participate in exercise (10, 12). Consequently, this lack of knowledge by sport coaches and teachers may result in young people with T1D losing their enthusiasm to participate in sport and exercise, which could potentially lead to them feeling ‘left out’ and ultimately leading to a development of a sedentary lifestyle behaviour.

Increasingly, the impact of exercise and diabetes care is being integrated into the clinical care and this has been systematically done by some teams globally (13, 14). However, there are no T1D exercise educational resources currently available in Australia to support the coaching community. Previous studies have reinforced that adolescents with T1D and their parents have repeatedly voiced the need for the development of these resources for them to feel supported during PA, especially during community sport (11, 12, 15). Hence, the overarching aim of this study was to identify the required resources needed to upskill coaches on T1D to improve their knowledge, understanding and confidence in coaching young people with T1D.

## Materials and methods

### Study design

A qualitative descriptive research design was employed for this study. Additionally, this study is part of larger study that utilised a participatory action research (PAR) methodology. This approach was employed as it involved both researchers and participants working in partnership to understand a problematic situation and improve it through knowledge production. This methodology is recognised as an important tool in health research to lessen the gap between perception and attitudes towards health and illness held by health professionals and lay people (16). Furthermore, this approach has been chosen because consumers are the central focus.

To ensure the needs of the community are met, online semi-structured interviews were conducted with different groups of people to collect the required information that will be used to guide the development of educational resources for community sport coaches. The study was approved by the Child and Adolescent Health Service Human Research Ethics Committee, Western Australia, and written informed consent was obtained from all participants prior to their participation in the study.

### Participants

A stratified purposive sample of participants were recruited through the Western Australian Children’s Diabetes Database and the Department of Local Government, Sport and Cultural Industries. The study recruited participants across three categories: i) young people living with T1D who participate/participated in community sport; ii) parents of young people living with T1D and iii) sports coaches who coach or are involved in community sport. In addition, our study aimed to recruit representatives of young people with T1D across the following age groups: children aged 8 to 11 years, adolescents aged 12 to 16 years and young adults aged 17 to 21 years. The recruitment of sports coaches included those from a range of sports and reflected the most popular sports played by young people living in Western Australia (2). Participants were excluded from the study if they: i) had insufficient English language skills that prevented them from giving informed consent; ii) or were unable to engage proficiently in an online interview.

### Interviews & procedures

A semi-structured interview schedule, with prompts to facilitate discussion, was developed by the research team. The interview questions were developed to include questions: i) for young people living with T1D and parents that focused on: a) their experience and interactions with community sport coaches regarding T1D; b) the preferred information on T1D for inclusion and c) the preferred media format of the educational resource and; ii) for community sports coaches which focused on: a) their level of confidence and previous experience in coaching a player with a medical condition; b) the preferred information about a player living with T1D for sports coaches in a community setting and c) the preferred media format of the educational resource. An interview guide/script for each of the three group of participants is presented in **Appendix A**.

The one-on-one interviews were completed online via Zoom at the workplace and were facilitated by one of the three research team members (1^st^ author: RL – Female; (BSc(Hons)), 2^nd^ author: AR – Female; (RN, MRes) and 3^rd^ author: JO – Female; (Grad Cert Public Health, BSc)) who are experienced in qualitative research and conducting interviews. RL and JO were research assistants, and AR was a clinical research nurse. All interviewers have worked within T1D research for multiple years and did not have any prior relationship with any of the participants. All interview sessions were video- and audio-recorded for transcription, with each interview discussion taking approximately 30 minutes. No field notes were taken during the interview and no interviews were repeated.

### Data analysis

Two authors (RL and ST) transcribed the audio-recorded interviews and cross-checked each transcript for accuracy. Transcripts were not returned to participants for comment. Data analysis was undertaken after the completion of five participants from each of the three categories and continued concurrently until no new codes emerged. All data was entered into NVivo software (QSR International). Two experienced researchers (RL and ST) used a thematic approached to analyse the data separately, establishing codes and themes from the data (17). Trustworthiness of the analysis process was achieved through i) note keeping and creating mind maps to demonstrate the interaction of the code which assisted in theme development (17). All data are reported in line with the consolidated criteria for reporting qualitative research guidelines (18).

## Results

Thirty-two participants, which included sixteen young people living with T1D (six males and ten females), eight parents and eight coaches were recruited. No participants withdrew from the study. The mean age and HbA1c level for the young people living with T1D was 13.9 ± 4.2 years and 7.6 ± 1.1%, respectively. All participants were pump and continuous glucose monitor (CGM) users. The interviews lasted between 15-44 minutes (mean 29.5 minutes) and took place between April 2021 to April 2022. The sports coaches that were interviewed were either the main or lead head coach of their respective club sports which included soccer, volleyball, hockey, basketball, gymnastics, swimming, and a kid’s holiday program. A summary of the participant demographic characteristics is presented in **Table 1**.

**Table 1:** Baseline demographic characteristics of study cohort.

	Children Aged 8 to 11 years	Adolescents Aged 12 to 16 years	Young Adults Aged 17 to 21 years	Parents	Sports Coaches
Total (n)	6	5	5	8	8
Males/Females (n)	1/5	3/2	2/3	2/6	3/5
Age (years)	9.92 ± 0.89	14.40 ± 0.96	19.70 ± 1.86	Not Applicable	Not Applicable
HbA1c (%)	7.87 ± 0.95	8.00 ± 1.34	7.04 ± 1.06	Not Applicable	Not Applicable
T1D Duration (years)	3.63 ± 2.04	5.13 ± 3.72	11.07 ± 5.56	Not Applicable	Not Applicable
Socio-Economic Indexes for Areas (n)					
* Quintile 1 (most disadvantaged)*				Not Applicable	Not Applicable
* Quintile 2*	1			Not Applicable	Not Applicable
* Quintile 3*	1		1	Not Applicable	Not Applicable
* Quintile 4*		1	1	Not Applicable	Not Applicable
* Quintile 5 (least disadvantaged)*	4	4	3	Not Applicable	Not Applicable
Type of sport coached (n)					
* Basketball*					1
* GymNot Applicablestics*					2
* Hockey*					1
* Kid’s Holiday Program*					1
* Soccer*					1
* Swimming*					1
* Volleyball*					1

Qualitative analysis identified themes within each group of participants, and these are discussed below. Additionally, all participants were asked about the specific information they wanted to include in the resource and their ideas for the resource format. This information will help guide the resource development.

### Themes identified by young people *‘Lack of understanding and needing help to stay safe’*

Young people identified that their sport coaches lacked an understanding about T1D and because of this, coaches did not appreciate how difficult T1D was to manage during sport. For example, *“At one point I had a low and they didn’t understand that my blood sugar was low but they thought that I was feeling kind of low, (as in) not very happy.”* – Adolescent #1. Additionally, young people described one of the main challenges they faced during sport was the ability to treat hypoglycaemia effectively and return to their sport in a timely manner, “*I don’t think the head coach fully understood if I was low. ‘cause if I was low, I would have to check it in between the sets and if I didn’t have enough time then I’ll literally have to chuck my stuff down and start rowing again. The break was two minutes and I’d have to try and squeeze a jellybean in that time if I’m low, it was quite stressful.”-* Young adult #3. Young people commented that it would be helpful if sport coaches used appropriate language to check in with players throughout training and games to remind players that they might need to check their blood glucose level or check how they are feeling. When asked, players identified phrases such as “*how are your numbers?”* or “*do you know what your levels are?”*

### Themes identified by parents ‘*Lack of knowledge and lack of confidence*’

Most parents felt that their child’s sports coaches lacked knowledge regarding T1D management and lacked confidence in how to treat hypoglycaemia and hyperglycaemia. This was the main reason that parents felt they needed to attend training sessions and games, *“The coach was scared and worried to the point that we always attended not only just the games, but training sessions. It was a case of if either my husband or I wasn’t there then [child] couldn’t play.” –* Parent #1. Parents expressed that they felt there was a lack of trust with sports coaches and therefore they could not leave their child. This lack of trust was due to several reasons including: a lack of communication, a lack of care/concern, or simply a lack of education about T1D and most of the time, parents had to create their own resources to give to coaches, “*It was very much an education piece so we gave the coaches a complete action plan of how to manage [child] for her blood sugar level management*” – Parent #6.

### Themes identified by coaches ‘*Clear understanding of responsibility*’

The theme of responsibility was identified by most sports coaches. Some coaches felt happy to take full responsibility for the child living with T1D while in their care, however, others felt this could be too demanding, especially considering some coaches are unpaid volunteers or parents. Additionally, coaches who were responsible across a number of children within a class felt it was difficult to focus all their attention on one child. Coaches identified that it was necessary to have clear communication from the parents about responsibility to determine the level of involvement from sports coaches during sport, “*Our job is to make sure we’re aware of it and work with the parents to make sure that’s it’s managed properly*” – Hockey coach. *“I think as long as the parents are comfortable sharing those things and generally they are, they want us to be able to take care of their kids as best as we can, but it just a little bit of a grey area for us at least to know how, and how many questions we can ask versus what’s really required for us to coach the child*” – Gymnastic coach. It was also evident that sports coaches lacked knowledge and wanted to know more about T1D so they could be equipped with the correct information on how to best support their players living with T1D during sport, *“So if their sugar levels drop really low, what do we do in that case? How do we know that that’s happening and what course of action and action plan should we have in place to make sure those children are safe?”* – Gymnastic coach.

With regards to how the resources will be presented upon development, each of the groups were asked directly about what information should be included in the resource and the preferred format.

Young people identified that the top three were signs and symptoms of hypoglycaemia and hyperglycaemia, being able to recognise a child’s change in behaviour and information and education surrounding T1D. Resource format suggestions included an A4 piece of paper, a video/s and a poster.

Parents noted that it was important to include in the resource i) signs and symptoms, ii) parent’s contact information for emergencies and iii) a procedure on how to treat hypoglycaemia. The formatting suggestions from parents were a fact sheet, palm cards and a poster. Parents also commented that the resources should have a simple layout with easy-to-understand language, and some suggested the resources should be laminated.

Sports coaches identified that signs and symptoms were also important to include in the resources. Additionally, sports coaches wanted to know the steps on how to respond in an emergency and the treatment options available. For format ideas, sport coaches noted that a PDF document, pocket cards or a 1-page action plan were good formats to suit a sporting environment. Majority of coaches commented that the resource needs to be quick to read and small enough to fit with their other coaching resources. Some coaches suggested an online course might be helpful, however others felt this may be too demanding and could be difficult for some coaches to complete as they were often unpaid volunteers.

It is also important to note that all three groups expressed the need for signs and symptoms of hypoglycaemia and hyperglycaemia to be included in the resource.

## Discussion

The aim of our study was to identify the information required to upskill coaches on T1D to improve their knowledge, understanding and confidence in coaching young people living with T1D in a community sport setting. This information has been used to develop educational resources for community sport coaches in the preferred formats. The interviews with young people living with T1D, parents and coaches have provided a wide perspective on information and has reinforced some of the challenges young people living with T1D face when playing community sport. Given that previous studies have identified the gap in T1D resources for sport settings (10, 12, 15, 19), our study is the first study to investigate what information is required to improve the knowledge of sport coaches to help young people living with T1D exercise safely and will assist with the development of the necessary resources to fill this gap.

The interview data from young people demonstrated that they felt their sports coaches lacked understanding around T1D and this lack of education resulted in a lack of support during sport. These same concerns have been expressed by other young people living with T1D in sport settings where young people and parents reported a lack of knowledge and understanding, especially amongst physical education teachers (12, 15). Despite them being aware of a person’s T1D diagnosis, parents and young people felt their physical education teachers didn’t know enough about how T1D can affect sporting performance. Our population identified mixed responses regarding the level of involvement they wanted from their coach. In our study, the younger players preferred their coaches checking on them as it made them feel safer. However, it has been reported that this can also cause a certain level of frustration by being constantly asked if they were alright and doing well during their sporting activities (12). A plausible explanation for this contrasting opinion may be due to the difference in ideologies of the participants from different age groups. In this present study, the young players who preferred the constant check-ins provided by their coaches were between the ages of 8 to 11 years and viewed the check-ins as support and care shown by their sport coaches. However, in Ryninks et al. (12) study, the participants were older (mean age of 14.5 years) and have been living with T1D for a longer duration as compared to our cohort. These differences in age and T1D duration could explain the level of frustration experienced due to the higher level of maturity and understanding surrounding their T1D management during sport and exercise. Additionally, the older aged players identified that they would rather be left alone to manage their own diabetes, as they tend to take on more responsibility for their management and relied less on their parents (12), which was similarly noted in our cohort.

Additionally, the differences in study findings observed between our present study and Ryninks et al. (12) study, which was conducted in the United Kingdom, may also be attributed to differences in confounding environmental factors present within each specific study populations. These environmental factors that may differ between the countries could include: i) the content and level of general education surrounding T1D management and specific education around T1D management during sport and exercise; ii) the sporting climate around sport and exercise within the respective communities and; iii) the level of engagement and participation in community sports by youths living with T1D. Consequently, these varying differences that exist currently may limit the generalisability of these educational resources on an international scale at this time. However, given that the development of these resources is still in its early stages, our intentions are to firstly address the needs and demands of these resources specific to the population and climate within Western Australia. Upon the successful implementation within the community in Western Australia, we will be aiming, in the long-term, to collaborate with external stakeholders and professionals both nationally and internationally, to implement these resources across Australia and the world, respectively. This stage of the project will be critical to allow for the generalisability of the resources worldwide given the individual and population differences that globally which include: i) content and level of T1D education; ii) community sport demographic and climate; and to a certain extent; iii) units of measurement and language. The parents in our study expressed their lack of confidence for sports coaches related to the lack of knowledge around T1D. Due to this lack of confidence, parents felt the need to be present at training sessions and games to lay a watchful eye on their child. These sentiments towards exercise and T1D have been similarly documented in previous studies (15, 20). Quirk et al. (15) explored parents’ perceptions of PA in children living with T1D and found that parents were reluctant to give responsibility to other people such as sport coaches due to the lack of specialised and skilled staff members. In our study, parents have expressed that if sport coaches or physical education teachers were trained on how to deal with diabetes, it will make them feel more comfortable and they would be more willing to leave their child alone during exercise and sport. This further highlights the need to increase sport coaches’ knowledge around T1D during PA performance.

It was evident that the sports coaches who were interviewed, lacked adequate knowledge surrounding T1D. The development of the educational resources may improve the level of knowledge of sports coaches and give them the appropriate tools to best manage their players with T1D. It Is difficult, however, to determine who the responsibility ultimately falls on. Interviews with sports coaches revealed that they would like open communication between parents and themselves to establish what expectations both parents and sport coaches have regarding the roles and responsibility of their child during sport. Hence, a key component of the educational resources that will be developed may help facilitate this discussion.

**Figure 1:**
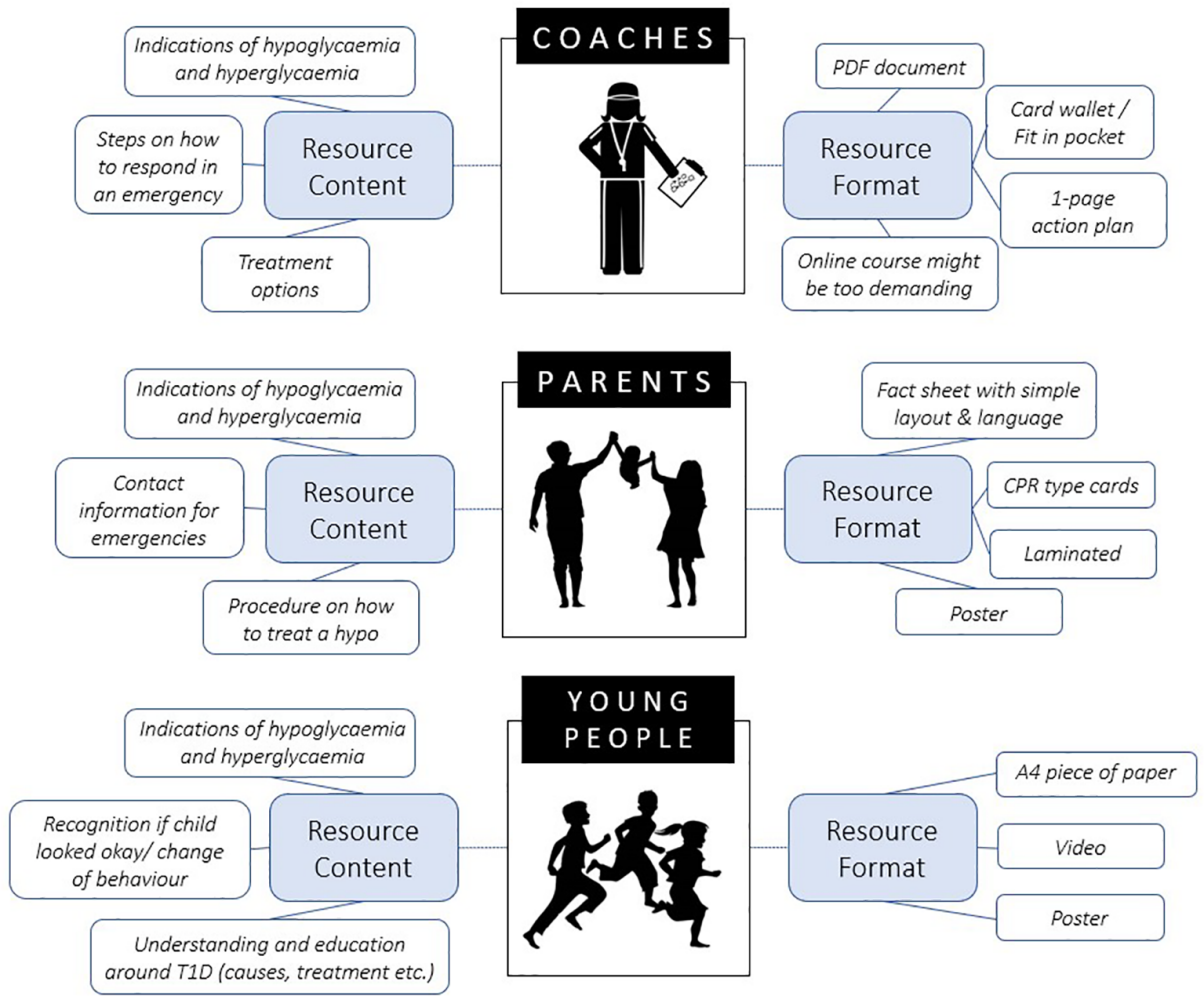
Resource content and format suggestions from each group to be developed for sport coaches.

With regards to the desired format for the educational resources, similar ideas were shared amongst the three participant groups (**Figure 1**), which ranged from a poster, A4 document or video. More specifically, and given that community sport coaches will be primary end-users of these resources, it was critical that we determined the desired format that sports coaches deemed appropriate, informative and practical to ensure that the resources will be utilised. Hence, based on the interviews conducted with the sports coaches in our study, the outstanding format idea was a simple resource that is small enough to fit with their other coaching equipment. A similar type of resource format has been previously identified for exercise and asthma, where coaches favoured a pocket guide (21).

Despite the focus of this present study being around community sport, the lack of knowledge surrounding T1D and exercise has been documented to extend into other sport settings which include physical education staff in school settings (12, 15, 20).Thus, this highlights the lack of education about T1D and exercise in the wider community and may plausibly be other avenues whereby the resources could be utilised in the future to educate and increase knowledge relating to T1D and exercise (12, 15, 22).

### Strengths and limitations

A key strength of our qualitative study is the stratified sampling and recruitment of participants from three age groups along with sport coaches from different sporting backgrounds. This ensured a wide variety of information was collected and allowed us to capture experiences that may be age-dependent or sport-dependent. This positive engagement helped us collect a holistic range of information required to aid in the development of the resources.

A potential limitation of our study could be the lack of exploration of experiences from young people with T1D who do not take part in community sport. Further studies could explore the attitudes and experiences of this population to see whether their reasons were similar to concerns shared by our cohort.

Although not a limitation, the sports coaches we interviewed were amateur level community sport coaches and their experiences coaching and requirements from a resource may be different from a coach for elite level athletes, which may be due to the access elite level coaches have to infrastructure and assistance from health care professionals within a team setting. Hence, future studies could look at interviewing coaches at a higher level to determine if their needs in a resource and knowledge of T1D is different to that of community sports coaches from our study population.

Given that most participants recruited in our study resided in quintiles 4 and 5, which indicate postcodes of least disadvantage as calculated via the Socio-Economic Indexes for Areas (SEIFA) (23), we acknowledge that this is a possible limitation and future studies should attempt to recruit participants across all areas to provide a greater representational spectrum to allow for a wider scope of information to be assessed.

## Conclusion

Our study is the first study to investigate the attitudes of young people with T1D, parents and sports coaches towards T1D management in a community sport setting. The interviews with all three groups identified that sports coaches lack adequate knowledge about T1D and that there is a lack of educational resources currently available to meet this need, Despite the benefits that existing educational resources have shown in the delivery of exercise programs, similar findings have been reported in previous research which showed parents and young people strongly expressed a lack of available resources for both T1D and other health conditions within the community (12, 15, 21, 24, 25). Consequently, the concerns expressed by parents and young people with T1D about the lack of current T1D exercise educational resources along with the participants in our study cohort reinforce the importance of developing educational resources to fill the gap in knowledge in sports coaches. Furthermore, the resources could potentially be used to support members of the wider sporting community such as teachers, physical education teachers and individuals who support young people with T1D during physical activity.

## Data availability statement

The raw data supporting the conclusions of this article will be made available by the authors, without undue reservation.

## Ethics statement

The studies involving humans were approved by Child and Adolescent Health Service Human Research Ethics Committee, Western Australia. The studies were conducted in accordance with the local legislation and institutional requirements. Written informed consent for participation in this study was provided by the participants’ legal guardians/next of kin.

## Author contributions

RL: Data curation, Formal Analysis, Writing – original draft, Writing – review & editing. AR: Data curation, Formal Analysis, Writing – review & editing. JO: Conceptualization, Data curation, Methodology, Writing – review & editing. VS: Conceptualization, Writing – review & editing. HR: Conceptualization, Project administration, Writing – review & editing. ED: Writing – review & editing. ST: Data curation, Formal Analysis, Writing – original draft, Writing – review & editing.
