# Supplementary material for: Developing type 1 diabetes resources: a qualitative study to identify resources needed to upskill and support community sport coaches
Source: Front Clin Diabetes Healthc. 2023 Nov 1;4:1284783. doi: 10.3389/fcdhc.2023.1284783 (PMC10646303; doi:10.3389/fcdhc.2023.1284783)
Supplement: Supplementary file 1 [file DataSheet_1.docx]

**Appendix A. Interview guide/script for players (young children with T1D), parents and sports coaches.**

*Interview Script – Players (Young Children with T1D)*

1. Tell me about the sport you play.

Prompts: what sports you play and how long you’ve played them?

1. Does having type 1 diabetes affect how you play or enjoy playing sport? If yes, how?

Prompts: Challenges? Hypos? Having to suspend the pump?

1. What have your interactions with your coaches been regarding your diabetes?

Prompts: do you tell them about your diabetes? Do they understand what you’re doing when you’re doing it? Do they understand or do you feel like they treat you differently to other players? Was the interaction initiated by you/them/family member?

1. What do you think coaches need to know about players with diabetes?

Prompts: Why/ why not?

1. What would you like the resource to look like?

Prompts: what would you be happy for your coach to use? Do you want it to be something that could be seen or accessed by team members as well as the coach, or something more discreet that others wouldn’t see them using?

1. Do you have any final comments you wish to add?

*Interview Script – Parents*

1. What is your experience of your child playing sport?

Prompts: Does it differ to their experience? How long have they played? What age did they start?

1. Tell me about the way your child discloses their diabetes to others.

Prompts: do they tell their coach/teammates? Why/why not? What are some of the experiences that have led to this? Did they find out about the diabetes from another means other than yourself/your child? How do you feel about others (including their coach) knowing?

1. What have been some of the good and bad experiences in sport for your child and their diabetes, in terms of a social perspective?

Prompts: How have teammates responded/reacted?

1. What information do you think a coach should know about a player with type 1 diabetes?

Prompts: Have you ever given your child’s coach any instructions? Why/why not? Was it helpful?

1. What are your expectations of someone coaching your child?

Prompts: would you expect them to assist/recognise when they are having a hypo?

1. Do you have any final comments you wish to add?

*Interview Script – Sports Coaches*

1. Tell me about your sport and sporting club.

Prompts: How long have you been coaching? Are you a volunteer or professional coach? Does your club require medical forms upon registration?

1. Have you ever coached a player with:
   - 1. Type 1 diabetes?
     2. A different medical condition/chronic disease? If yes, what:

[If Yes to A) and/or B)]:

1. What was your experience with this player?

Prompts: How did you find out they had the condition? Did they provide you with any information about this condition? Did you ever have to assist with the player’s diabetes management/care? Who was responsible for this player during training/the game? What was important for you to know about Type 1 Diabetes/their medical condition (if a different chronic disease)?

[If No to both A) and B)]:

1. How do you feel about coaching someone with type 1 diabetes?

Prompts: Responsibilities? Dealing with parents? Handover of responsibility from parent to coach and vice versa?

1. If a player discloses to you that they have a medical condition, what would you like to know?

Prompts: how would you like this provided? Video? Poster? Clipboard card? Would you use it?

1. Do you have any final comments you wish to add?
2. Conclusion:

Thank you for your time this evening and for putting your hands up to contribute to developing a resource. This will hopefully help our type 1 kids feel more supported and encouraged to participate in sport, put parents’ minds at ease and help coaches to be able to assist the kids living with type 1 diabetes.
